# Mammalian Metallothionein-3: New Functional and Structural Insights

**DOI:** 10.3390/ijms18061117

**Published:** 2017-05-24

**Authors:** Milan Vašák, Gabriele Meloni

**Affiliations:** 1 Department of Chemistry B, University of Zürich, Winterthurerstrasse 190, CH-8057 Zürich, Switzerland; 2 Department of Chemistry and Biochemistry, University of Texas at Dallas, 800 W Campbell Road, Richardson, TX 75080-3021, USA

**Keywords:** metallothionein-3, copper, zinc, metal-thiolate clusters, neurodegeneration, reactive oxygen species, metal homeostasis, amyloid

## Abstract

Metallothionein-3 (MT-3), a member of the mammalian metallothionein (MT) family, is mainly expressed in the central nervous system (CNS). MT-3 possesses a unique neuronal growth inhibitory activity, and the levels of this intra- and extracellularly occurring metalloprotein are markedly diminished in the brain of patients affected by a number of metal-linked neurodegenerative disorders, including Alzheimer’s disease (AD). In these pathologies, the redox cycling of copper, accompanied by the production of reactive oxygen species (ROS), plays a key role in the neuronal toxicity. Although MT-3 shares the metal-thiolate clusters with the well-characterized MT-1 and MT-2, it shows distinct biological, structural and chemical properties. Owing to its anti-oxidant properties and modulator function not only for Zn, but also for Cu in the extra- and intracellular space, MT-3, but not MT-1/MT-2, protects neuronal cells from the toxicity of various Cu(II)-bound amyloids. In recent years, the roles of zinc dynamics and MT-3 function in neurodegeneration are slowly emerging. This short review focuses on the recent developments regarding the chemistry and biology of MT-3.

## 1. Introduction

Aging, a major risk factor for neurodegenerative disorders, is accompanied with structural, chemical, functional, neuropsychological, and genetic changes, with increased susceptibility to diseases and cognitive impairments [1]. In the normal brain, a high concentration of essential transition metal ions such as zinc, copper, and iron is present. The homeostasis of these transition metals is tightly regulated and essential for brain physiology [2,3]. In physiological conditions, micromolar concentrations of zinc and copper are actively released from neurons during neurotransmission processes into pre- and postsynaptic clefts. However, in dysregulated metal metabolism, present in neurodegenerative disorders, the ill-binding of copper and zinc to the disease specific amyloidogenic peptides or proteins correlates with deposition of amyloid fibrils. Among the most prominent examples are the Aβ peptides in Alzheimer (AD), α-synuclein in Parkinson (PD), prion protein in prion deposits typical of Creutzfeldt–Jakob disease (CJD), and superoxide dismutase-1 (SOD-1) in amyotrophic lateral sclerosis (ALS). In these diseases, copper, due to its reactivity with molecular oxygen (O_2_), generates neurotoxic reactive oxygen species (ROS) such as superoxide, hydrogen peroxide, and hydroxyl radicals [2,3].

In the central nervous system (CNS), the natural metal chelator metallothionein-3 (MT-3) represents one of the major players in copper and zinc homeostasis. This protein occurs intra- and extracellularly and was found down-regulated in AD [4]. However, in contrast to the almost exclusive expression of MT-3 in the CNS [5], the expression of MT-1/-2 isoforms are found in almost all organs. Besides the differences in the expression pattern, the control of MT-3 expression also differs from the MT-1/-2 isoforms. Thus, in marked contrast to the induction of MT-1/-2 biosynthesis by a range of factors including glucocorticoids, cytokines, reactive oxygen species, and metal ions [6], the expression of MT-3 is unresponsive to these inducers. However, the *MT-3* gene was identified as hypoxia-inducible in several human tissues. Here the induction of *MT-3* in cultured human astrocytes by hypoxia suggested that the protein may protect the brain from hypoxic damage [7]. Furthermore, a similar hypoxic induction of the *MT-3* gene in human adipocytes implied that the protein may protect adipocytes from hypoxic damage [8]. In other studies, the question of whether the MT-3 and MT-1 isoforms possess different biological properties has been addressed using cultured kidney cells that allowed constitutive expression of both isoforms. The studies revealed that under zinc deficient conditions MT-3, but not MT-1 inhibited the cell growth [9]. The neuronal growth inhibitory activity of MT-3 appears to be the most prominent biological property of this protein. In this regard, an extracellular addition of MT-3, but not MT-1/-2 counteract the ability of AD brain extract to stimulate survival and neuritic sprouting of cultured neurons [10,11]. Both the growth inhibitory activity and the protective effect of solely MT-3 from the toxic effect of amyloid peptide Aβ_1–40_ [10] have been linked to its possible role in the pathogenesis of AD. However, the bioactivity and the protective role of MT-3 in AD are functionally unrelated. An increased interest in the physiological and pathophysiological processes in the human brain brought about an increased research into the understanding of the structure and biology of MT-3. In recent years, the list of MT-3 functions suggesting its role not only in the CNS but also outside of this organ, is steadily increasing. At present, there are a few reviews on record dealing with the MT-1 through MT-3 isoforms [12,13,14,15,16,17,18,19,20,21,22]. This review focuses on the recent advances in our knowledge regarding the structural/chemical and functional properties of MT-3 in metal related biological processes.

## 2. Structural and Chemical Properties of Metallothionein-3

**Figure 1:**
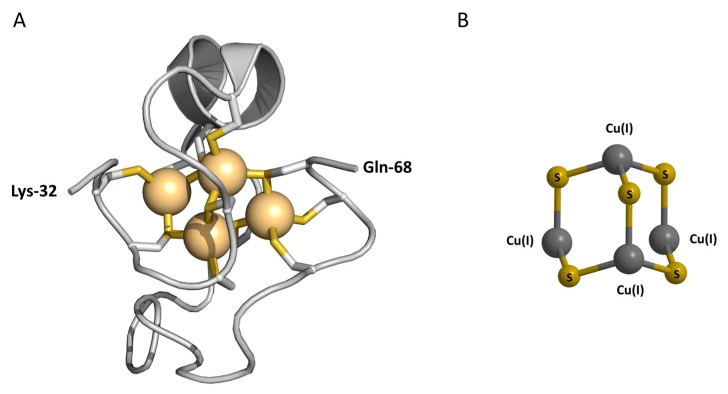
Metal-thiolate clusters in metallothionein-3. (**A**) The NMR solution structure of the Cd_4_-α-domain of human Cd_7_MT-3. The Cd(II) ions are shown as light-orange spheres connected to the protein backbone by cysteine thiolate ligands. The model was generated with PyMOL using the Protein Data Bank coordinates of 2FJ5 [28]; (**B**) model of the Cu(I)_4_CysS_5_ cluster, derived from the spectroscopic characterization of Cu(I)_4_,Zn_4_MT-3 [29,30]. Metal ions are shown as dark gray spheres connected to cysteine sulfur ligands (yellow).

As recent publications cover the protective role of MT-3 in AD, and its possible role in lead detoxification in the human brain, we wish to briefly summarize the current knowledge about the structure and reactivity of the protein required to discuss these new reports. Since the bioactivity of MT-3 in neuronal assays has been established for the native protein, containing both copper and zinc ions, and for the recombinant human Zn_7_MT-3 [4,11,23], the structural features of both metalloforms have been investigated (Table 1). The structural studies on recombinant M^II^_7_MT-3 revealed that, like the M^II^_7_MT-1/-2 isoforms, the seven divalent metal ions are tetrahedrally coordinated through the array of 20 conserved cysteines forming two metal-thiolate clusters, i.e., M^II^_3_(CysS)_9_ and M^II^_4_(CysS)_11_. A M^II^_3_(CysS)_9_ metal-cluster is located in the N-terminal β-domain (residues 1–31) and a M^II^_4_(CysS)_11_ cluster is located in the C-terminal α-domain (residues 32–68) of M^II^_7_MT-3 [24,25]. In these clusters, unprecedented dynamic processes exist. A fast exchange between conformational cluster substates and very slow exchange processes between configurational cluster substates in the β-domain differentiate MT-3 from other isoforms [26]. As a result, only the 3D structure of the C-terminal α-domain of mouse and human ^113^Cd_7_MT-3, containing an adamantane-like Cd_4_Cys_11_ cluster, could be determined by NMR measurements [27,28]. The structure of this domain reveals a peptide fold and cluster organization very similar to that found in mammalian Cd_7_MT-1/-2 (Figure 1A).

Native MT-3, as isolated from human [4] and bovine [31] brains, contains four Cu(I) and between three and four Zn(II) ions. The isolated Cu(I)_4_Zn_3–4_MT-3 is a monomeric protein that is stable in air. Extended X-ray absorption fine structure (EXAFS) studies on the native protein revealed the presence of two homometallic clusters, a Cu(I)_4_–thiolate cluster and a Zn_3–4_–thiolate cluster [31]. In contrast to tetrahedrally coordinated Zn(II) ions, Cu(I) ions are diagonally and/or trigonally coordinated by two or three cysteine ligands [32]. The localization of both clusters in the protein structure was established by the spectroscopic and immunochemical studies of Cu(I)_4_,Zn_4_MT-3. Thus, the Cu(I)_4_–thiolate cluster is located in the N-terminal β-domain and the Zn_4_–thiolate cluster in the C-terminal α-domain [22,33]. Based on the spectroscopic and luminescence properties of air-stable Cu(I)_4_,Zn_4_MT-3 (Table 1), generated in the aerobic reaction between Zn_7_MT-3 and free Cu(II), the presence of a Cu(I)_4_S_5−x_ cluster containing five reduced thiolates and two disulfide bonds has been postulated (Figure 1B) [29,30]. It may be noted that the adamantane-like {M_4_S_6_} polyhedron is the most frequently observed species in copper-thiolate chemistry. Therefore, by analogy with inorganic model complexes, besides the reduced cysteine thiolates the participation of a disulfide bridge sulfur (indicated by x) in the Cu(I) coordination cannot be excluded.

A fundamental question about all metalloproteins is what determinants control which metals they bind in vivo. In some cases, metals are delivered to the metalloproteins by specialized metallochaperones. However, for most metalloproteins, a critical factor is the availability of the appropriate metal species in the buffered pools in the cell. In a number of studies, the *Escherichia coli* expression system was used to address the question regarding the in vivo metal-binding abilities of a number of MTs [34]. In this regard, the particular MT was first recombinantly expressed in *E. coli* cultures enriched with Zn(II), Cd(II), or Cu(II). Subsequently, ZnMT preparations were then titrated in vitro with Cd(II) or Cu(I) ions and the generated metalloforms analyzed. A comparison of the *E. coli* generated metalloforms with those formed in vitro was taken to indicate the metal binding preference of the particular MT. Based on such studies carried out on MT-3, it has been concluded that this MT isoform exhibits a marked Cu–thionein character, with a high tendency to coordinate Cu(I) ions [35]. In all these studies, a high degree of metal selectivity is discussed by the authors in terms of inherent properties of MTs. A critical assessment of this approach in assigning the metal selectivity of MTs in vivo has been given [35]. In this work, it has been put forward that all metals in the cell are present buffered to given concentrations, due to their binding to other proteins and low molecular weight compounds. The metal binding preference of a particular MT in *E. coli* cultures implies that the metal selectivity reflects its structural properties. However, the *E. coli* cytosol still contains other proteins and low molecular weight compounds contributing to the metal buffering. Consequently, the exact maintenance of the ratios of buffered metal ions will dictate the correct metal incorporation into a protein. Overall, it would appear that the binding of the correct metal into the protein structure might be regulated by adjusting the ratios of buffered metal ions to different location in various cell types [36]. A similar conclusion has been drawn from computational studies in which, in the absence of metallochaperons, the local metal concentrations were found critical for the metal specificity of proteins [37]. Although the enhanced copper-thionein character of MT-3 over MT-1/-2 may still, in part, reflect subtle differences between the two sequences and consequently their structural properties [38], it would appear that the reported metal binding preference of various MTs in *E. coli* cultures may not exactly reflect the buffered metal concentrations present in different mammalian cell types where the MT biosynthesis occurs.

## 3. Metallothionein-3 in Metal-Linked Neurodegenerative Disorders

The protective role of MT-3 from AD pathology has been implicated based on the finding that the protein (a) is down-regulated in AD, (b) shows extracellular growth inhibitory activity and antagonizes the ability of AD brain extract to stimulate survival and neuritic sprouting, and (c) protects, by an unknown mechanism, the neuronal cells from the toxic effect of amyloid peptide Aβ_1–40_ [4,5,10,11,39]. In an AD brain, Aβ forms amyloid plaques in specific regions in the brain, which have been considered a key element in its pathogenesis [40,41,42]. Furthermore, a large body of evidence connects the metal-induced aggregation of Aβ by transition metal ions copper and zinc and accompanied oxidative stress with the disease progression [43]. In contrast to redox-inert zinc, redox-active copper aberrantly bound to amyloidogenic Aβ peptides can activate molecular oxygen, resulting in the production of ROS through Fenton and Haber–Weiss reactions [44]. In view of the observation that extracellular Zn_7_MT-3 protects the neuronal cells from the toxic effect of amyloid peptide Aβ_1–40_ [10,45], an interaction of free Cu(II) and that bound in Cu(II)–Aβ_1–40_ with Zn_7_MT-3 has been found responsible for this protective effect [30]. The spectroscopic and mass spectrometric studies on the interaction between free Cu(II) and human Zn_7_MT-3 revealed that concomitant with the Cu(II) reduction to Cu(I) by the thiolate ligands the monovalent copper is bound to the protein. As a result, an air-stable Cu(I)_4_Zn_4_MT-3 species is formed cooperatively in the β-domain together with two intramolecular disulfide bonds and the release of three originally bound Zn(II) ions. Furthermore, the Cu(II)-mediated hydroxyl radical production was fully abolished, indicating that the protein, through the formation of the redox-inert Cu(I)_4_Zn_4_MT-3 structure, efficiently scavenges the free Cu(II) ions [30]. In the follow up studies, the question as to the reported protective effect of human Zn_7_MT-3 against Aβ_1–40_ toxicity in neuronal cell culture [10] has been addressed. The toxicity of Aβ_1–40_ stems mainly from the formed Cu(II)–Aβ_1–40_ complex in the extracellular space and its interaction with molecular oxygen, whereby the ROS are generated. In vitro studies of the interaction between Cu(II)–Aβ_1–40_ and Zn_7_MT-3 established that the protein can efficiently remove Cu(II) from both the soluble and insoluble aggregates of Cu(II)–Aβ_1–40_. The products of this reaction are the already discussed air-stable Cu(I)_4_Zn_4_MT-3 species and the redox-inert Zn(II)–Aβ_1–40_ complex. In addition, the metal-swap between Zn_7_MT-3 and Cu(II)–Aβ_1–40_ fully abolishes the Cu(II)-mediated ROS production. This protective effect against the ROS toxicity has been demonstrated not only by in vitro experiments but also in human neuroblastoma cell cultures [46]. The mechanism of exchange of metal ions between Zn_7_MT-3 and Cu(II)–Aβ_1–40_ was also investigated by spectroscopy and transmission electron microscopy [47]. The studies revealed that metal ion exchange occurs via free Cu(II) on a time scale of seconds to minutes and that metal ion exchange induces time-dependent amyloidogenic structural and morphological changes in Aβ_1–40_ on a time scale of hours. The morphological changes were primarily due to the binding of Zn(II) to Aβ_1–40_ aggregates [48]. Taken together, these studies corroborate a possible beneficial role of endogenous MT-3 in protecting from the AD etiology. In vivo studies, investigating the function of MT-3 in double transgenic mice presenting AD pathology (Tg2576 mouse AD model), support this role [49]. In a mouse AD model, APP increases death rate and affects behavioral phenotype. The absence of MT-3 partially reversed the APP-induced mortality and changes in the behavior of females. Furthermore, the amyloid plaques formation and/or APP expression in the cortex and hippocampus of MT-3 deficient females were decreased. Interestingly, MT-3 absence, on the one hand, substantially decreased APP in brain homogenates from female mice and, on the other hand, shows a tendency to increase copper levels in both genders [49].

In the later studies, the protective effect of the human Zn_7_MT-2A isoform against Cu(II)–Aβ_1–40_ toxicity was also investigated and compared with that of Zn_7_MT-3 [50]. The studies showed that MT-2A can protect against copper-induced Aβ aggregation and neurotoxicity. However, in contrast to the original studies employing Zn_7_MT-3, in which the formation of the air stable Cu(I)_4_Zn_4_MT-3 species was found crucial for the protective effect [46], the underlying reaction between Cu(II)–Aβ_1–40_ and Zn_7_MT-2A has been interpreted using the chemical characteristics of the fully reduced Cu(I)_10_MT-2A and Cu(I)_12_MT-3 species. However, as the reduction of Cu(II) in Cu(II)–Aβ_1–40_ to Cu(I) is accomplished by the thiolate ligands of Zn_7_MTs, the formation of fully reduced Cu(I)_10_MT-2A and Cu(I)_12_MT-3 in the oxidizing extracellular environment is unlikely. This conclusion is supported by the mass spectrometric demonstration that the cysteine thiolates in Zn_7_MT-3 can reduce up to 8 Cu(II) [30] concomitant with the formation of intramolecular disulfides. Thus, while the stepwise reduction of the first 4 Cu(II) equivalents confirmed the cooperative formation of the air-stable Cu(I)4Zn4MT-3 species, the reductions of the following Cu(II) equivalents resulted in the simultaneous presence of a number of air-sensitive Cu(I)MT-3 species [30]. These studies signify the importance of Cu(I)_4_Zn_4_MT-3 for its protective effect in AD. By contrast, in the studies of the interaction between Cu(II)–Aβ_1–40_ and Zn_7_MT-2A, the direct metal swap between Zn(II), coordinated by thiolate ligands in Zn_7_MT-2A and Cu(II), coordinated by nitrogen and oxygen ligands in Cu(II)–Aβ_1–40_, has been implicated and the importance of metal affinities for the protective effect stressed [50]. However, the assumed direct metal swap between Cu(II)–Aβ_1–40_ and Zn_7_MT-2A cannot occur without the preceding Cu(II) reduction to Cu(I) by cysteine ligands. Accordingly, the differences in affinities are not essential for the protective effect, but rather the long-term stability of partially oxidized Cu(I),ZnMT-2A formed upon Cu(II) reduction and its removal from Cu(II)–Aβ_1–40_ in the oxidizing extracellular space. Thus, to gain an insight into the protective effect of Zn_7_MT-2A the redox stability of different Cu(I)_x_, Zn_y_MT-2A species, generated upon aerobic Cu(II) reduction, should be addressed.

The amyloid hypothesis in AD considers the Aβ production and deposition as the trigger of this neurodegenerative disorder [51]. So far, the protective effect of Zn_7_MT-3 from the toxicity of the Cu(II) containing Aβ_1–40_ and Aβ_1–42_ peptides has been described [46]. However, the proteolytic cleavage of APP by β- and γ-secretase yields several other isoforms of the Aβ peptides. β-Secretase, the β-site APP-cleaving enzyme 1 (BACE1), is an aspartyl protease [52,53,54], while γ-secretase is a multiprotein complex consisting of at least four essential components (presenilin, nicastrin, Aph-1, and Pen-2) necessary for full enzymatic activity [55]. The APP cleavage by β- and γ-secretase gives rise to Aβ_1–42_ and Aβ isoforms ranging from Aβ_1–40_ down to Aβ_1–17_ [56]. In addition, β-secretase cleavage together with α-secretase cleavage yield several short Aβ peptides [55]. In this context, the increased concentrations of Aβ_1–16_ have been found in sporadic AD (SAD) and familial AD (FAD) compared to non-demented controls.

Apart from Aβ with aspartate (Asp1), as the first amino acid in Aβ_1−x_, several N-truncated and modified Aβ peptides have been found in the brains of AD patients. However, to extract and identify the exact levels of the various N-truncated Aβ variants in post-mortem brains is difficult as many factors—such as antibody specificities, extraction protocols and brain areas studied—can influence an analysis [57]. Nevertheless, there is general agreement that plaque-born peptides harbor high amounts of N-truncated Aβ, especially that starting with Phe4. As revealed by detailed analyses of Aβ isoform pattern in the cerebellum, cortex, and hippocampus of FAD subjects, SAD subjects and non-demented controls, the dominating Aβ isoforms in AD brains are Aβ_4–42_, Aβ_1–40_, and Aβ_1–42_ [58,59]. The first three amino acids (Phe-Arg-His) of N-truncated Aβ_4–42_ give rise to the H_2_N-Xaa–Yaa–His– motif which enables a high affinity Cu(II) binding [60], already reported in human serum albumin (HAS) [61]. Recently, the question regarding the Aβ_4–42_ affinity toward Cu(II) ions has been addressed [62]. By using the Aβ_4–16_ peptide as a model to fully characterize the Cu(II) binding properties, it was demonstrated that the Phe-Arg-His sequence of Aβ_4–16_ stoichiometrically binds Cu(II) with a conditional *K*_d_ value of 30 fM at pH 7.4 (a conditional *K*_d_ is defined as the *K*_d_ at pH 7.4). The determined affinity is more than three orders of magnitude higher than that reported for Aβ_1−x_ peptides (conditional *K*_d_ ∼1 × 10^−10^ M) [63]. Consequently, Aβ_4–16_ can compete successfully with Aβ_1−x_ peptides for Cu(II) ions. Furthermore, Aβ_4–40/42_ forms fibers twice as fast as Aβ_1–42_ with a very different morphology, forming bundles of very short amyloid rods. The generation of ROS by Cu(II)–Aβ_4–16_, monitoring the hydroxyl radical production, revealed that the N-truncated Cu(II)–Aβ_4–16_ and Cu(II)–Aβ_4–42_ species exhibit low levels of hydroxyl radical production compared to Cu(II)–Aβ_1–42_. The results are in agreement with voltammetric experiments in which low level of redox activity of the Cu(II)–Aβ_4–16_ complex was determined. Thus, Cu(II) in Cu(II)–Aβ_4–16_ could be oxidized irreversibly to Cu(III), but could not be reduced to Cu(I) [62].

In view of the latter studies, the question has been addressed whether the N-terminal Cu(II)–Aβ_4–16_ complex is fully resistant to the copper/zinc swap with Zn_7_MT-3 [64]. In marked contrast to Cu(II)–Aβ_1–16_, no copper can be extracted from the N-truncated Cu(II)–Aβ_4–16_ complex by Zn_7_MT-3. A reverse swap experiment, where apo–Aβ_4–16_ was added to the preformed Cu(I)/Zn(II)MT-3 complex, was also conducted. Here the absence of Cu(I) transfer from MT-3 to the peptide reflects different affinities of thiolate vs. oxygen and nitrogen ligands for Cu(I) ions. Conversely, addition of Cu(II) ions to a mixture of Zn_7_MT-3 and apo–Aβ_4–16_ resulted in the partition of copper between these biomolecules. From these studies, it has been concluded that Aβ_4–42_ and Zn_7_MT-3 may play parallel roles in synaptic copper clearance in different synaptic settings [64].

Other naturally N-terminally truncated Aβ possessing the copper binding motif H_2_N-Xaa–Yaa–His– is the Aβ_11–40/42_ peptide which starts with the residue Glu11. This peptide is found in the cerebrospinal fluid and has a similar abundance to Aβ_1–42_, constituting one-fifth of the plaque load [65]. A recent publication reported for Cu(II)–Aβ_11–42_ *K*_d_ of a 34 fM at pH 7.4 [66], which is similar to 30 fM reported for Cu(II)–Aβ_4–42_. In this work, no studies regarding the redox stability of the Cu(II)–Aβ_11–42_ complex and the peptide reactivity with Cu(II)–Aβ_1–x_ or Zn_7_MT-3 have been conducted [66]. Nevertheless, the N-truncated Aβ_11–42_ peptide may show a similar reactivity to that reported for to the Aβ_4–42_ peptide (see above). However, although Aβ_11–42_ species with an intact N terminus could be detected, the majority of peptides truncated at the N-terminal residue Glu11 are cyclized to pyroglutamate to generate pGlu-Aβ_11–42_ [67,68]. This peptide modification blocks the Cu(II) coordination site.

**Figure 2:**
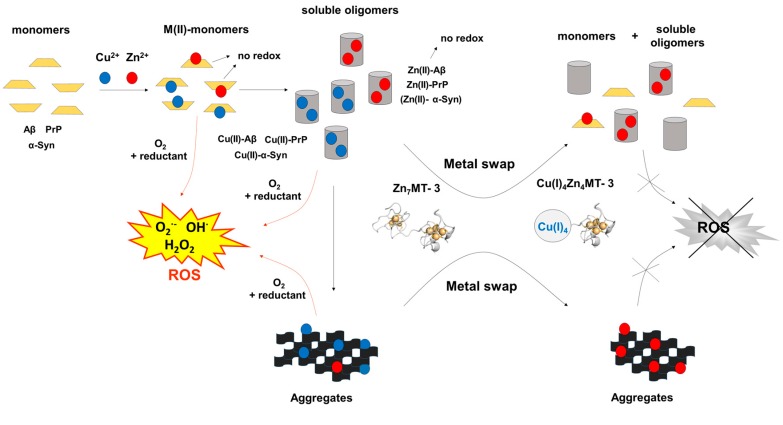
Metal-swap reactions and Cu(II) redox-silencing by Zn_7_MT-3 in neurodegenerative disorders. The protective effect of human Zn_7_MT-3 (with the structure of β-domain modeled by that of rat Zn_2_Cd_5_MT-2 [77]) from the copper-mediated toxicity in AD, PD and prion diseases is summarized. The metal swap between Zn_7_MT-3 and the disease specific amyloidogenic Cu(II)–Aβ_1–40_ peptide [46], the Cu(II)–α-Syn [75] and Cu(II)–PrP proteins [76] abolishes the ROS production and the related cellular toxicity. In this process, Cu(II) is reduced by the protein thiolates concomitant with its binding into the N-terminal β-domain, forming the Cu(I)_4_Zn_4_MT-3 species and the non-redox-active Zn(II)–Aβ_1–40_, Zn(II)–α-Syn and Zn(II)–PrP. In Cu(I)_4_Zn_4_MT-3 an air-stable Cu(I)_4_-thiolate cluster and two disulfide bonds are present the N-terminal β-domain.

Apart from the discovered protective effect of MT-3 in AD, its role in other metal-linked neurodegenerative disorders like PD and prion disease has also been investigated. Similarly to AD, the aberrant binding of Cu(II) to the amylogenic protein α-synuclein (α-Syn) in PD [69,70,71] and the prion protein in CJD [2] has deleterious effects in these diseases. These include the Cu(II)-mediated ROS production and contribution to the formation of oligomers and fibrils. PD is characterized by the loss of dopaminergic neurons and the formation of intracellular inclusions known as Lewy bodies, composed mainly of aggregated α-Syn in neurons of *substantia nigra pars compacta* [72]. The formation of Cu(II)–α-Syn contributes to the progression of PD. In the pathogenesis of prion diseases, the structural conversion of a natively folded prion protein (PrP^C^) [73], into a misfolded (PrP^Sc^) is the key molecular event [2,74]. While PrP^C^ is mainly a helical monomeric protein, PrP^Sc^ is oligomeric and rich in β-sheet structure [2]. The binding of up to six Cu(II) to the histidine residues present in the disordered part of PrP^C^ structure plays an important role in its conversion to PrP^Sc^ and thus the disease progression. The studies of the interaction between Zn_7_MT-3 and Cu(II)-containing α-Syn and prion protein in vitro revealed that Zn_7_MT-3, through copper subtraction and the formation of air-stable Cu(I)_4_Zn_4_MT-3, plays a protective role [75,76]. Taken together, as the nature of the Cu(II) binding sites in Aβ, α-Syn, and prion proteins substantially differ, the observed protective effect of Zn_7_MT-3 against Cu(II) toxicity suggests that the protein may play a general protective role in metal-linked neurodegenerative disorders (Figure 2).

## 4. Toxicological and Neuropathological Aspects of Lead (Pb) Toxicity in Relation to Mammalian Metallothioneins

The biosynthesis of most predominant mammalian isoforms MT-1/-2, but not of MT-3 is upregulated in response to different stimuli, especially heavy metals, but also oxidative stress or stress hormones (glucocorticoids). High cysteine content of MT-1/-2 (ca. 30%) confers them with a high capacity to bind essential (Zn(II) and Cu(I)) and toxic (Cd(II), Ag(I), and Hg(II)) heavy metal ions in vivo and in vitro. The exposure of humans or experimental animals to these metal ions lead to an induction of MT-1/-2 biosynthesis and the formation of MT-1/-2 metalloforms in which the corresponding metal ion is bound. The mechanism of the metal-mediated induction of MT-1/-2 biosynthesis is discussed below. The role of MT-1/-2 in the heavy metal detoxification has been established first for Cd(II) [78,79,80,81,82]. In recent years, there is an increased interest in understanding of the toxicity of lead (Pb), a ubiquitous naturally occurring toxic metal. Pb-induced toxic effects can manifest in several organs but the brain and kidney are primary targets. Compelling evidence is emerging that Pb exposure, particularly in early-life, may cause neurodegeneration in later life stages. The sporadic nature of AD argues for an environmental link that may drive AD pathogenesis. In rodents, exposure to Pb during brain development predetermined the expression and regulation of the APP, its amyloidogenic Aβ product and other AD-related genes in old age [83]. Life time whole-body occupational Pb exposure has been recently shown to be a risk factor for PD [84]. Since the formation of Lewy bodies in PD required MT-1/-2, the interaction between α-Syn and MT-1/-2 has been established using MT-null and wild-type cell lines [85]. A direct interaction between α-Syn and MT-1/-2 was confirmed by antibody pull-down assay [86]. However, a similar study using the MT-3 isoform has not been undertaken.

By analogy to the MT-1/-2 induction by Cd(II), the formation of mammalian Pb(II)MT-1/-2 in a similar detoxification process has been anticipated. However, so far no Pb(II)-containing MTs have been isolated and characterized from natural sources. Nevertheless, the in vitro characterization of the Pb(II)-containing MT-1/-2 and MT-3 isoforms has been reported [87,88]. Prior to the discussion of the structural features of these Pb(II)-containing MTs, the current knowledge regarding the nature of low molecular weight Pb(II)-binding proteins, obtained upon the Pb(II) administration to experimental animals, will be briefly discussed. Apart from these proteins, the heme pathway enzyme δ-aminolevulinic acid dehydratase (ALAD) isoforms (240–280 kDa proteins), have attracted the greatest attention as the enzymes are both inhibited and induced by lead (reviewed in [89]).

From a number of studies, the nature of low molecular weight proteins appears to be uncertain as the occurrence of either low molecular weight lead-binding proteins or MT-like proteins have been reported [89]. At this point, it should be noted that in all instances, no full characterization of these proteins has been carried out. Lead-binding proteins are a series of low molecular weight proteins (10 to 12 kDa) which have the capacity to bind and sequester lead in a nontoxic form in several organs (kidney, brain, lung, liver, and erythrocyte). These proteins are rich in aspartate and glutamate amino acids and possess a dissociation constant *K*_d_ for Pb(II) on the order of 10^−8^ M and appear to normally bind zinc [90]. The occurrence of low molecular weight Pb(II)-binding protein(s) with a large percentage of cysteine and a greater UV absorbance at 254 than at 280 nm, features diagnostic of a MT, have also been reported [91]. In this regard, it has been shown that depending on the conditions of Pb(II) administration to experimental animals the gel filtration fractions containing only ZnMT, both PbMT and ZnMT metalloforms, or the metal mixed Pb,ZnMT form have been identified [89]. These observations can be explain, in part, by the underlying mechanism of the detoxification process described for Cd(II) [92]. A hallmark of the promoters/enhancers of most *MT* genes are short DNA sequence motifs termed metal response elements (MREs); many also harbor antioxidant response elements (AREs) and glucocorticoid response elements (GREs) [93]. Transcriptional induction of *MT-1/-2* genes is mediated by the metal-regulatory transcription factor 1 (MTF-1) [92,94]. This protein is essential not only for zinc-induced transcription of *MT-1/-2* genes, but also for their induction by other heavy metals like cadmium and copper and/or oxidative stress [92]. In the induction process, MTF-1, upon activation with zinc displaced from zinc-saturated Zn_7_MT-1/-2 in the cytosol by cadmium or copper binding, translocate from the cytosol to the nucleus where it binds to promoter proximal MREs [94,95]. Lead, similarly to cadmium and copper, will also displace Zn(II) from Zn_7_MT-1/-2 and activate MTF-1. Consequently, depending on the experimental conditions the newly synthetized thionein (the metal-free form of MT-1/-2) may then bind with different metal ions reflecting the concentration of buffered metals available at folding (see above). It may be noted that although similar MREs and other DNA sequence motifs were also identified in the *MT-3* gene, the MT-3 biosynthesis was unresponsive to these inducers [39].

The spectroscopic/biochemical studies on the in vitro interaction of Pb(II) with MT-2 and MT-3 have been carried out and the Pb(II)-MT forms characterized [87,88]. In the structure of M^II^_7_MTs, the divalent metal ions Zn(II) and/or Cd(II) are tetrahedrally coordinated by four cysteine sulfurs. Recent structural studies aimed at the understanding of Pb(II) binding to thiol-rich structural proteins containing zinc-binding sites. The studies showed that although zinc is bound tetrahedrally, Pb(II) shows a clear preference for a three-coordinate Pb(II)-S_3_ mode. In the latter structure, Pb(II) is coordinated by three sulfurs in a trigonal pyramidal geometry with the Pb 6s^2^ lone-pair electrons occupying the axial position [96]. It has been concluded, moreover, that Pb(II) assumes a preferred coordination mode of Pb-S_3_ even when an additional thiolate ligand is available for binding. The interaction of Pb(II) with MT-2 has been studied by combination of spectroscopic and computational methods [87]. By contrast to Zn(II) and Cd(II), the binding of Pb(II) results in the formation of two different Pb_7_MT-2 complexes depending on the pH employed. The first complex, designated Pb_7_MT-2(I), is formed under neutral conditions and the second, Pb_7_MT-2(II), under weakly acidic (pH 4.5) conditions, The characterization of Pb_7_MT-2(I) revealed that all seven Pb(II) ions are trigonally coordinated by three cysteine sulfurs (Pb–S_3_ mode) and that no metal-ligand ring in the α- or β-domain is present. The marked structural differences to the cluster structure in Zn(II)- and Cd(II)-containing M^II^_7_MTs results from the Pb 6s^2^ lone-pair electrons, which disrupt the tetrahedral coordination by occupying the axial position. In the case of Pb_7_MT-2(II), completely different binding modes from Pb_7_MT-2(I) exist with all cysteine ligands coordinating in the terminal form. These binding modes include the trigonal pyramidal Pb–S_3_ mode, the distorted trigonal pyramidal Pb–S_2_O_1_ mode in the α-domain, and the distorted quadrilateral pyramidal Pb–S_3_O_1_ in the β-domain.

The binding of Pb(II) and Zn(II) to MT-3 has been studied by isothermal titration calorimetry (ITC), a technique capable of measuring the thermodynamics of metal binding [88]. Anaerobic ITC measurements of Pb(II) displacing Zn(II) from Zn_7_MT-3 and experiments in which EDTA was used to chelate metal ions from both Pb_7_MT-3 and Zn_7_MT-3 revealed that (i) MT-3 binds Pb(II) with a higher affinity than it does Zn(II) and that Pb(II) displaces bound Zn(II) in Zn_7_MT-3 and that (ii) EDTA extracted both metal ions from Zn_7_MT-3 and Pb_7_MT-3 in a tri-phasic process, indicating that they bind to the protein in three populations with different binding thermodynamics. These data indicate that Pb(II) binding to both apoMT-3 and Zn_7_MT-3 is thermodynamically favorable [88]. Overall, despite the number of toxicological studies over many years, the in vivo interaction of MTs with Pb(II) is not entirely clear.

## 5. Concluding Remarks

Since the initial discovery of MT-3 in normal human brains by Uchida et al. 1991 [4], the functional role of this metalloprotein has been investigated intensively. MT-3 is involved in homeostasis of essential transition metals zinc and copper in the brain. The amyloid-forming diseases such as AD, PD, ALS, and prion (CJD) represent a group of neurodegenerative disorders that affect both animals and humans. In these diseases, the role of metal ions, especially copper and zinc, is the subject of intense research. Their involvement in protein misfolding, aggregation and in the generation of reactive oxygen species have been shown. The structural and biological studies also show that these metal ions not only bind to the disease specific amyloidogenic peptides or proteins with high affinity, but also modify their biochemical properties, making them important players in the progression of the disease. The protective effect of MT-3 from the copper toxicity has been demonstrated for AD, PD, and prion diseases (see above). The neuroprotective effect of MT-3 in the pathogenesis of ALS has also been established. In this work, MT-3 prevents the loss of motor neurons of ALS model mice and prolongs the life span, even when the administration is started at the time of onset [97]. Furthermore, recent biological data reveal that MT-3 contributes to neuronal and astrocytic cell death through zinc release [98] and that its interaction with actin modulates c-Abl signaling in astrocytes; c-Abl is a member of the nonreceptor tyrosine kinase family [99]. In addition, the observed developed obesity in male MT3-null mice is due likely to abnormal leptin signaling in the hypothalamus [100]. Apart from the CNS, the MT-3 expression is also observed in a few non-CNS areas, including the pancreas [101], prostate, testis, and tongue [102]. In this regard, more recent studies on the zinc-rich insulin producing islet β-cells suggest a role for MT-3 in streptozotocin (STZ)-induced islet cell death and consequent hyperglycemia [103]. Thus, it has been demonstrated that MT-3 may through PDE3a (phosphodiesterase 3A) play a key role in zinc dyshomeostasis and cell death in STZ-treated islets. Overall, the list of MT-3 functions, suggesting its role not only in the CNS, but also outside of this organ, is steadily increasing.

## Figures and Tables

**Table 1:** Electronic absorption and luminescence properties of Zn_7_MT-3, Cd_7_MT-3, and Cu(I)4Zn4MT-3 [26,29,30]. ^a^ ligand-to-metal-charge-transfer; ^b^ lifetime.

MT-3 Form	Absorption	Luminescence (77 K)					
		High-Energy Band			Low-Energy Band		
	1st LMCT ^a^ Band (nm)	λ_em_ (nm)	τ ^b^ (μs)	λ_max_ Excitation Spectra (nm)	λ_em_ (nm)	τ (μs)	λ_max_ Excitation Spectra (nm)
Zn_7_MT-3	~230	-	-	-	-	-	-
Cd_7_MT-3	~250	-	-	-	-	-	-
Cu(I)_4_Zn_4_MT-3	~265	425	~40	envelope < 350	565	~135	~265; ~300

## Abbreviations

Aβ	Amyloid-beta
AD	Alzheimer’s Disease
ALAD	δ-aminolevulinic acid dehydratase
ALS	Amyotrophic lateral sclerosis
APP	Amyloid precursor protein
AREs	Antioxidant response elements
BACE	Beta-secretase
CJD	Creutzfeldt–Jakob disease
CNS	Central nervous system
EXAFS	Extended X-ray absorption fine structure
FAD	Familiar Alzheimer’s Disease
GREs	Glucocorticoid response elements
ITC	Isothermal Titration Calorimetry
MRE	Metal response elements
MT	Metallothionein
MTF-1	Metal-regulatory transcription factor 1
PD	Parkinson’s Disease
PrP	Prion Protein
PSEN1	Presenilin 1
PSEN2	Presenilin 1
SAD	Sporadic Alzheimer’s Disease
SOD	Superoxide Dismutase
α-Syn	Alpha-Synuclein
